# Asbestos, carbon nanotubes and the pleural mesothelium: a review of the hypothesis regarding the role of long fibre retention in the parietal pleura, inflammation and mesothelioma

**DOI:** 10.1186/1743-8977-7-5

**Published:** 2010-03-22

**Authors:** Ken Donaldson, Fiona A Murphy, Rodger Duffin, Craig A Poland

**Affiliations:** 1 University of Edinburgh, Centre for Inflammation Research, Queens Medical Research Institute, 47 Little France Crescent, Edinburgh, EH16 4TJ, UK

## Abstract

The unique hazard posed to the pleural mesothelium by asbestos has engendered concern in potential for a similar risk from high aspect ratio nanoparticles (HARN) such as carbon nanotubes. In the course of studying the potential impact of HARN on the pleura we have utilised the existing hypothesis regarding the role of the parietal pleura in the response to long fibres. This review seeks to synthesise our new data with multi-walled carbon nanotubes (CNT) with that hypothesis for the behaviour of long fibres in the lung and their retention in the parietal pleura leading to the initiation of inflammation and pleural pathology such as mesothelioma. We describe evidence that a fraction of all deposited particles reach the pleura and that a mechanism of particle clearance from the pleura exits, through stomata in the parietal pleura. We suggest that these stomata are the site of retention of long fibres which cannot negotiate them leading to inflammation and pleural pathology including mesothelioma. We cite thoracoscopic data to support the contention, as would be anticipated from the preceding, that the parietal pleura is the site of origin of pleural mesothelioma. This mechanism, if it finds support, has important implications for future research into the mesothelioma hazard from HARN and also for our current view of the origins of asbestos-initiated pleural mesothelioma and the common use of lung parenchymal asbestos fibre burden as a correlate of this tumour, which actually arises in the parietal pleura.

## Background

The experience with asbestos highlighted that high aspect ratio particles (fibres) pose an additional hazard to the lung beyond that produced by conventional compact particles and gave rise to the discipline of fibre toxicology. Over several decades up to the present, fibre toxicology has evolved a structure activity paradigm that explains the pathogenicity of fibres that is the most robust in particle toxicology. Whilst this paradigm explains the relationship between fibre characteristics and their pathogenicity, the exact sequence of events, following fibre deposition leading to a fibre-type hazard to the pleura and pleural mesothelium has not been clarified. In particular we poorly understand the mechanism whereby fibres seem to selectively deliver their dose to the parietal pleura, whilst the visceral pleura is not initially affected [1]. There has been no unifying hypothesis as to exactly how, within the normal understanding of particle clearance of particles out of the lungs, sustained fibre 'dose' is delivered to the parietal pleura sufficient to produce the distinctive profile of pleural effects associated with fibre exposure in man and animals. In this paper we advance a plausible hypothetical mechanism that emphasises translocation of a fraction of all deposited particles and fibres to the pleural space but the retention of only long fibres in the parietal pleura. This retention of fibre dose at the parietal pleura then serves as the driver that initiates mesothelial injury and inflammation that over time leads to pleural pathology, including mesothelioma. This mechanism is, we contend, generalisable to carbon nanotubes and potentially to other high aspect ratio nanoparticles (HARN) that are currently a cause for concern due to their asbestos-like morphology and which represent the driving stimulus for this work.

The application of this hypothesis to nanotubes arises from our initial work regarding the similarities in length-dependent mesothelial inflammogenicity of asbestos and carbon nanotubes in the peritoneal cavity [2]. The role of failed peritoneal and pleural cavity clearance of long fibres in their pathogenicity was advanced in its essential form by Kane and co-workers [3,4]. Boutin and co-workers [5-7] have also made essentially the same suggestion as regards asbestos and clearance from the parietal pleura in relation to human asbestos-related mesothelioma. We therefore fully recognise these intellectual precedents in our restatement and elaboration of the hypothesis here in relation to our work with long and short nanotubes in the pleural cavity [2]. This new work, which is mentioned to a small extent in this review, is being submitted for full peer-review elsewhere (Murphy, F., Poland, C.A., Ali-Boucetta, H., Al-Jamal K.T., Duffin, R., Nunes, A., Herrero, M-A., Mather, S. J., Bianco, A., Prato, M., Kostarelos, Donaldson, K. Long but not short nanotubes are retained in the pleural space initiating sustained mesothelial inflammation. Submitted for publication)This present review sets out the anatomical and pathophysiological background concerning the behaviour of particles and fibres in the pleural space and elaborates the evolving hypothesis that might explain how long fibres and long nanotubes might deliver 'dose' to the parietal pleura.

## Fibres and the pleural mesothelium

Particles tend to deliver their effects to the lung itself in the form of fibrosis or lung cancer. PM_10 _particles also affect susceptible populations, exacerbating existing airways disease and cardiovascular disease, probably via pro-inflammatory effects emanating from the lungs. The additional hazard posed by fibres relates to the mesothelial lining of the pleural cavity and to some extent the peritoneal cavity. Individuals exposed to asbestos demonstrate a wide range of pleural pathologies including pleural effusion (a build up of fluid within the pleural space), pleural fibrosis and pleural mesothelioma [8]. A variable, usually small, proportion of mesotheliomas developing in individuals exposed to asbestos arise in the peritoneal cavity, likely as a result of fibre translocation from the pleural cavity to the peritoneal cavity [9]. The mechanism of production of pleural mesothelioma is not well understood although various mechanisms have been advanced [10]. However some contact between fibres and mesothelial cells is a reasonable supposition (see below) and numerous studies have demonstrated effects such as genotoxicity [11] and pro-inflammatory effects [10] following exposure of mesothelial cells to asbestos and other fibres *in vitro*.

## The classical fibre pathogenicity structure:activity paradigm

**Figure 1:**
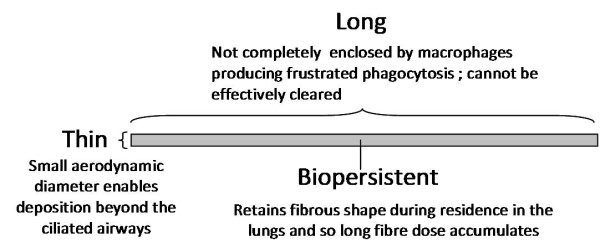
**Diagram illustrating a pathogenic fibre according to the pathogenicity paradigm and the role of particle characteristics**.

Several decades of fibre toxicology have lead to an over-arching fibre toxicology structure:activity paradigm involving length, diameter and biopersistence (reviewed in [12] Figure 1).

The fibre paradigm identifies the geometry of fibres as their most important toxicological characteristic and not the chemical make-up, except in so far as the composition makes a contribution to biopersistence (see later). This independence from composition is evident in the fact that the paradigm embraces fibres composed of diverse materials including amphibole and serpentine asbestos minerals, vitreous and ceramic fibres and an organic fibre (reviewed in [12]). Diameter is important because of the central role that fibre diameter plays in defining aerodynamic diameter (D_ae_) and the dependence of pulmonary deposition on D_ae _[13]. Clearance from beyond the ciliated airways is dominated by slow, macrophage-mediated clearance [14] and so fibres which deposit there have the potential to contribute most to build-up of dose. Length impacts little on D_ae _for thin fibres [15] except when length is sufficient to cause interception, a mechanism of particle deposition that is confined to fibres, involving the centre of gravity of the fibre following the airstream at a bifurcation whilst the tip of the fibre makes contact with the wall, resulting in deposition. The penetration of long fibres (>50 μm) beyond the ciliated airways is explicable on the basis that the aerodynamic diameter of a straight fibre is around 3 times its actual diameter [15]. This results from its alignment with the airflow as the fibres move aerodynamically through these tubes, aligned along the axis of the bronchial tree.

The evidence demonstrating that length is a key factor in pathogenicity of fibres comes from a number of sources but the best data are from experimental toxicological studies where it is possible to isolate length categories and assess their effects, unlike the mixed nature of human exposures. In the seventies Stanton carried out a large number of studies aimed at understanding the role of fibre characteristics in mesothelioma using implantation of fibres in gelatin, directly onto the pleural mesothelial surface. Although this is a highly artificial exposure, in a summary of these studies [16] Stanton identified that carcinogenicity was related to 'durable' fibres longer than 10 μm. In the study by Davis *et al*. [17] rats were exposed in a chamber to clouds with equal airborne mass concentration of either long amosite asbestos fibre or a short fibre amosite sample obtained from it by ball-milling. After lifetime exposure there was substantial tumour and fibrosis response in those rats exposed to the long amosite and but virtually no response in rats exposed to the short amosite. Adamson *et al*. used long and short crocidolite and following deposition in mouse lungs reported fibrosis [18] and proliferative responses [19] at the pleura with the long, but not the short samples. The mouse peritoneal cavity has been used as a model of direct mesothelial exposure and much greater toxic [20], inflammatory [21] and granuloma-generating [4] responses were evident in mice that were exposed to high doses of long fibres than was seen with shorter fibres. *In vitro *systems have also demonstrated the greater potency of long compared to short fibres in assays of pro-inflammatory and genotoxic activity [22-27].

Biopersistence and length interact in determining the clearance of long fibres from the lungs since long fibres may undergo dissolution which could result in complete dissolution, or most likely weakening of the fibre such that it undergoes breakage into shorter fibres, which can be more rapidly cleared than long fibres. The retention half-time (T_1/2_) of a compact, inert, respirable, tracer particle, or a short fibre, in the respiratory tract of a rat is commonly ~60 days [28]. However long fibres (> 20 μm) are more slowly cleared as they cannot be easily enclosed by macrophages [29] leading to frustrated phagocytosis (see below). Thus long fibres are more likely to accumulate in the lungs allowing the long fibre dose to build up. Long fibres that are composed of bio-soluble (non-biopersistent) structural components can undergo weakening and breakage in the lungs [30]. The knowledge described above lead to the evolution of the fibre pathogenicity paradigm show in Figure 1 highlighting that a pathogenic fibre is one that is long, thin and biopersistent.

## Carbon nanotubes and the classical fibre pathogenicity paradigm

**Figure 2:**
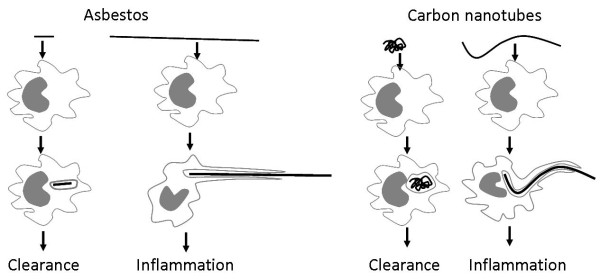
**The frustrated phagocytosis paradigm as it relates to long and short fibres of asbestos (left) and various forms of carbon nanotubes (right)**. When confronted by short asbestos fibres or tangled, compact carbon nanotube 'particles' the macrophage can enclose them and clear them. However the macrophage cannot extend itself sufficiently to enclose long asbestos or long nanotubes, resulting in incomplete or frustrated phagocytosis, which leads to inflammation.

**Figure 3:**
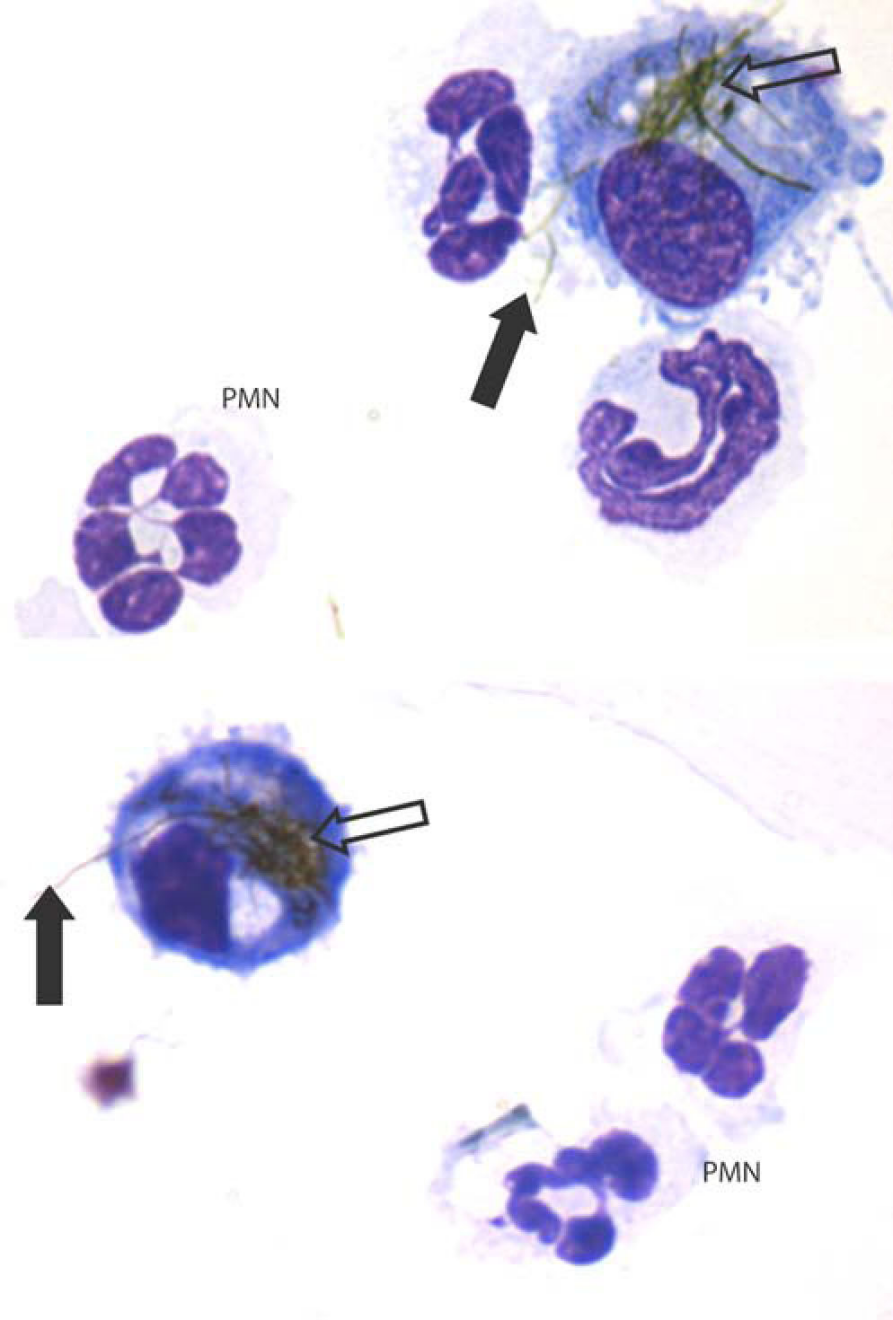
**Frustrated phagocytosis (arrows) and the associated acute inflammatory reaction in the bronchoalveolar lavage of mice whose lungs have been instilled with long nanotubes**. Aspiration of 50 μg of long fibrous multi-walled carbon nanotubes (CNT) into the lungs of C57BL/6 mice caused an acute inflammatory reaction at 24 hrs typified by a large influx of inflammatory neutrophils (PMN) into the bronchoalveolar lavage. CNT bundles and singlet fibres were seen both within macrophages (hollow arrow) and extending outside the macrophage in the process of incomplete or frustrated phagocytosis (black arrows). All images at taken at ×1000 magnification.

Carbon nanotubes (CNT) are one of the most important products of nanotechnology, representing significant investment and are already incorporated into a large number of products and this is likely to increase. However, the essentially fibrous structure of CNT has lead to concern that they might cause asbestos-like pathology in the lung and mesothelium [2,31]. Carbon nanotubes can exist as compact tangles of nanotubes that are essentially particles, or as longer, straighter 'fibres' and we would anticipate that the hazard from these two different forms of carbon nanotube would differ. Particle effects would be confined to the lungs as fibrosis and cancer whilst fibres, exemplified by asbestos, are known have the same types of pulmonary effect but to also affect the pleura. We previously carried out a study where we exposed the peritoneal mesothelium, as a convenient model for the pleural mesothelium, to carbon nanotubes to determine whether they showed an asbestos-like, length-dependent toxicity [2]. These studies revealed that carbon nanotubes in the form of long fibres showed a similar, or greater, propensity to produce inflammation and fibrosis in the peritoneal cavity, to that produced by long asbestos. In contrast neither short asbestos fibres nor short, tangled CNT caused any significant inflammation. One important underlying process in the toxicity of long fibres is the failure of the macrophage to completely enclose them- termed incomplete or 'frustrated' phagocytosis, which is a pro-inflammatory condition. Long carbon nanotubes very likely cause inflammation via this process when long enough, i.e. longer than about 15 μm [2]. Frustrated phagocytosis of long fibres as it likely applies to asbestos and carbon nanotubes is illustrated in Figures 2 and 3.

In terms of the fibre pathogenicity paradigm, it is possible for carbon nanotubes to be pathogenic by being thin, long and biopersistent but unlike other fibres it is also possible for CNT to exist in forms that do not comply with the paradigm for a pathogenic fibre. For example CNT can exist as short forms, and longer but tangled forms (see right side of Figure 2), neither of which would pose a problem to macrophages in terms of phagocytosis or clearance. Whilst singlet nanotubes are always thin they form tangles, ropes and wires of intertwined tubes and these can be thicker, although still likely to be thin enough to be respirable. However, in larger tangles and bundles the aerodynamic diameter may well increase beyond respirability. Graphene, the basic structural component of CNT is an exceedingly strong material [31] and so is likely to be biopersistent when the graphene is pristine, with few defects and underivatised, and that is suggested by our own data (in preparation). However CNT derivatised by some chemistries, with increased amounts of defects in the graphene structure, may be less biopersistent.

## Injection of fibres into the peritoneal cavity as a surrogate for fibre effects in the pleural cavity: the role of retention of long fibres in the peritoneal cavity in long fibre-induced inflammogenicity and fibrogenicity

**Figure 4:**
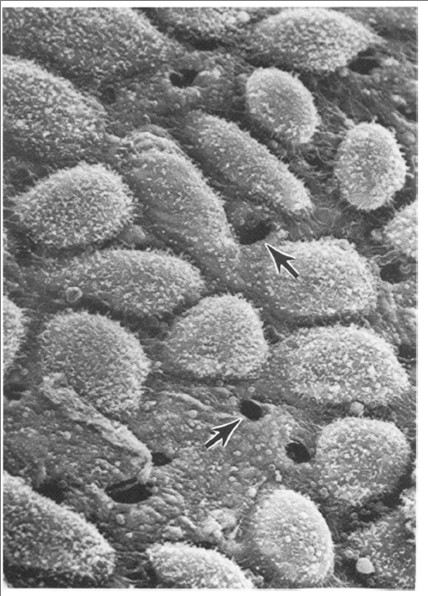
**SEM of the surface of the peritoneal face of the diaphragm of a mouse showing the stomata (arrows) reproduced with permission from Moalli et al **[4].

The peritoneal cavity and its viscera are covered by a mesothelium and this was recognised as a convenient surrogate for the pleural cavity mesothelium in fibre studies over 30 years ago. Subsequently asbestos fibres were found to produce inflammation [21] and mesotheliomas [32] in the peritoneal cavity following injection. Although the peritoneal cavity would not be expected to have evolved the efficient clearance mechanisms shown by the lungs, in fact it does have a system for removing particles. Instilled particles are rapidly drawn cranially in the lymph flow through the diaphragm to the parathymic lymph nodes [33]. This involves transit through the diaphragm via stomata which are pore like structures less than 10 μm in diameter (Figure 4) linking the peritoneal cavity to the underlying lymphatic capillaries and which were implicated in fibre effects by Kane and co-workers in 1987 [4].

**Figure 5:**
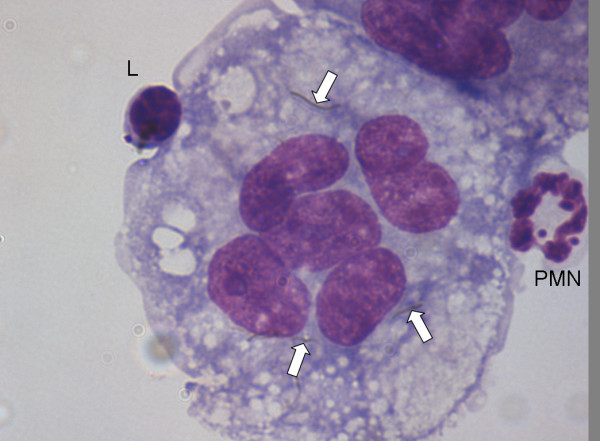
**Multinucleate giant cell lavaged from the peritoneal cavity of a mouse instilled with long carbon nanotubes**. CNT are visible in the cytoplasm (arrows); PMN = Poymorphonuclear Neutrophilic Leukocyte; L = lymphocyte (Magnification ×100).

**Figure 6:**
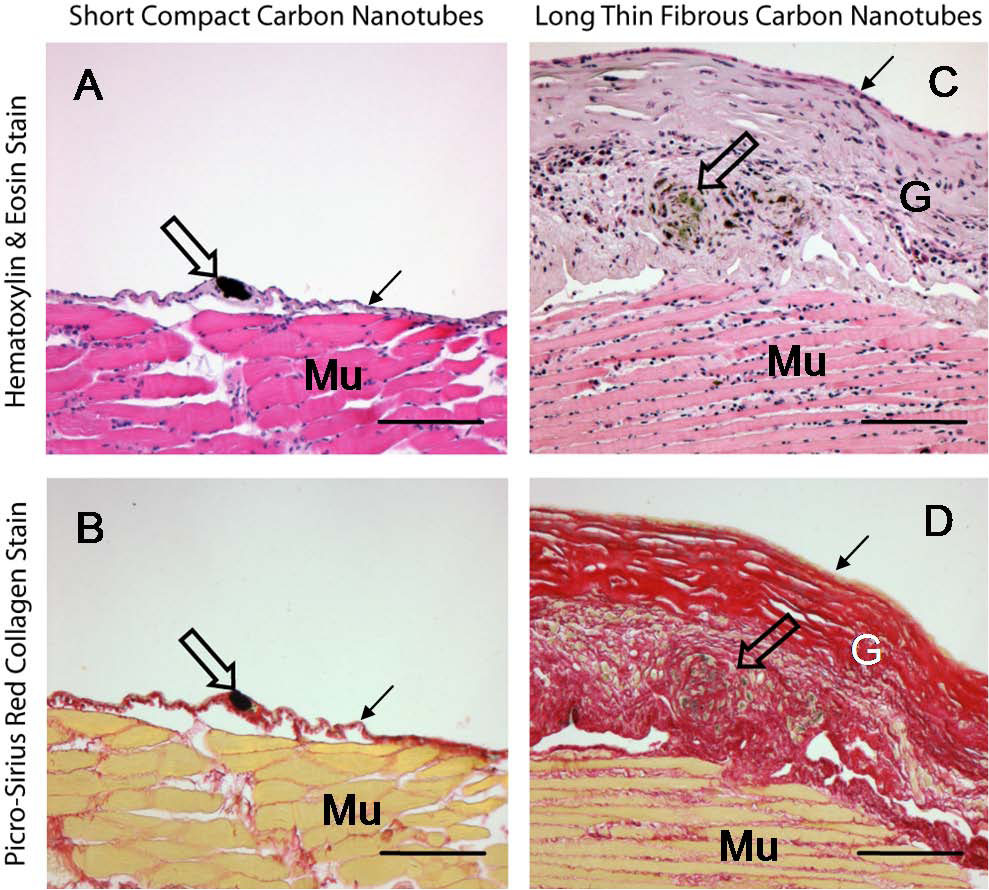
**Lesions on the peritoneal face of the diaphragm after intra-peritoneal injection of 2 forms of carbon nanotube differing in aspect ratio**. The figure shows sections through the peritoneal aspect of the diaphragm of C57BL/6 mice 6 months after intra-peritoneal injection of 10 μg of two separate forms of multi-walled carbon nanotube (CNT). Sections are stained with Haematoxylin & Eosin (panels A and C) or Picro-sirius red stain which stains collagen bright red (panels B and D). Mu = Muscle of diaphragm; G = granuloma; small arrows = mesothelium; large arrows = carbon nanotubes. C and D show a large granuloma sitting atop the muscle layer, caused by the presence of CNT in the form of long fibres (open arrows). A and B show the contrasting response to CNT in the form of tightly bound dense spherical aggregates (open arrows) which produces minimal tissue reaction. All images are taken at ×100 magnification bar = 100 μm.

Kane and co-workers [3] noted that long asbestos fibres accumulated preferentially at the peritoneal face of the diaphragm around the stomata since they could not be cleared through them due to their length. Kane *et al*. contended that retention of long fibres at the diaphragmatic mesothelial surface initiated inflammation, proliferation and granuloma formation. Short fibres did not cause this effect, easily exiting through the stomata. However, if short fibre were injected at such high dose that their sheer volume blocked the stomata this prevented clearance allowing retention, resulting in inflammation [3]. We confirmed that low dose exposure of the mouse peritoneal cavity to long multi walled carbon nanotubes (MWCNT) [34] resulted in accumulation of the long CNT at the diaphragm, suggesting that they are also too long or bulky to exit through the stomata. Retention of the long CNT in the peritoneal cavity initiated granulomas with classical foreign body giant cells in the peritoneal lavage (Figure 5) and in the granulomas (Figure 6) [2]. Short, tangled CNT were not retained and were never seen at the diaphragm or viscera and did not induce inflammation or granuloma formation, their absence from sections strongly suggesting that were cleared through the stomata.

Therefore, we would postulate that there are two important parts to the mechanism of the pro-inflammatory effects of long fibres in the peritoneal cavity, shared by both asbestos and long MWCNT

**i) **failure of long fibres to negotiate the diaphragmatic stomata with subsequent retention of the long fibre dose at the diaphragm; this contrasts with smaller particles which easily leave the peritoneal cavity through the diaphragmatic stomata to accumulate in the parathymic nodes [33],

**ii) **at the point where the long fibre dose accumulates at the peritoneal face of the diaphragm macrophages attempt to phagocytose the long fibres; they then undergo frustrated phagocytosis stimulating inflammation and mesothelial cell damage, leading to chronic inflammation and granuloma development [2].

The consequences of pro-inflammatory and fibrogenic effect of long fibre retention at the diaphragm were most evident in the extent of the granuloma/fibrosis response seen 6 months following instillation of 10 μg of long or short nanotubes (see [2] for a full description of the NT tang 2 and NT long 1 nanotubes used in this study). As Figure 6 shows, the presence of quite a large aggregate of short/tangled nanotubes produced very little tissue reaction (Figure 6A and 6B). In contrast, loose aggregates and singlet fibres of long nanotubes caused a florid granuloma response (Figure 6C and 6D). The multi-layered basketwork -like arrangement of largely acellular collagen in the granuloma is strongly reminiscent of the structure of an asbestos-induced pleural plaque (see later).

The issue of peritoneal mesothelioma arises and begs the question as to the extent of translocation of fibres from the pleural space to the peritoneal cavity. Little is known about this and it has never been quantified in man. The mere fact that far-and-away most mesotheliomas arise in the pleural cavity suggests that long fibres are retained there, so short fibres can reach the peritoneal cavity but, by virtue of their low pathogenicity, generally do not cause much harm there.

## Pleural structure and function

**Figure 7:**
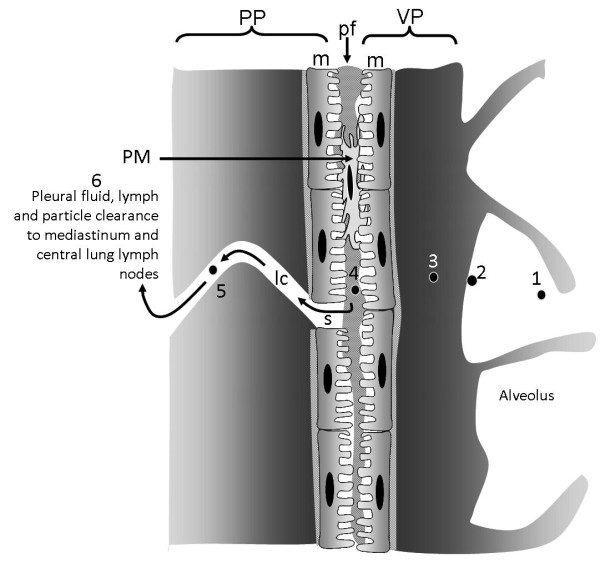
**Diagrammatic representation of the relationship between the visceral and parietal pleurae**. The visceral pleura (VP) and the parietal pleura (PP) are seen in close apposition separated by a pleural space that contains a small volume of pleural fluid (pf). Contact between the 2 pleurae is made via the mesothelial cell layers (m) on the surface of the parietal and visceral pleurae. Pleural macrophages (PM) are present in the pleural space. The rigid chest wall is tightly locked to the lungs by the adherence of the visceral pleura to the parietal pleura allowing movements of the chest wall caused by the action of the diaphragmatic muscle and intercostal muscle (IM) to expand and relax the lungs, allowing pulmonary inspiration and expiration. The pathway for particles to reach the pleural space is unknown but the path for an airborne particle (1) that deposits in the distal alveoli (2) is shown as it passes into the interstitium (3) enters the pleural space (4) and exits through a stoma in the parietal pleura (s) into a lymphatic capillary (lc, 5) to enter the lymph flow to the lymph nodes in the mediastinum and central lung.

Although the peritoneal cavity serves as a convenient model to study mesothelial impacts of fibres, the primary mesothelial target for inhaled fibres is the pleural mesothelium. The chest cavity, or pleural cavity is the cavity that surrounds the lungs and heart, comprising the ribs and associated muscles and connective tissue. This cavity is covered by the parietal pleura, which is attached to the chest wall and is covered by a continuous 'parietal' mesothelial cell layer. The lungs themselves are enclosed by the visceral pleura which is integral to the lung surface and which has a surface 'visceral' mesothelial layer. The tight fit of the lungs to the inside of the chest wall means that the two mesothelial layers are closely apposed and there is a thin space between them that contains the pleural fluid (Figure 7) and also a population of pleural macrophages.

**Figure 8:**
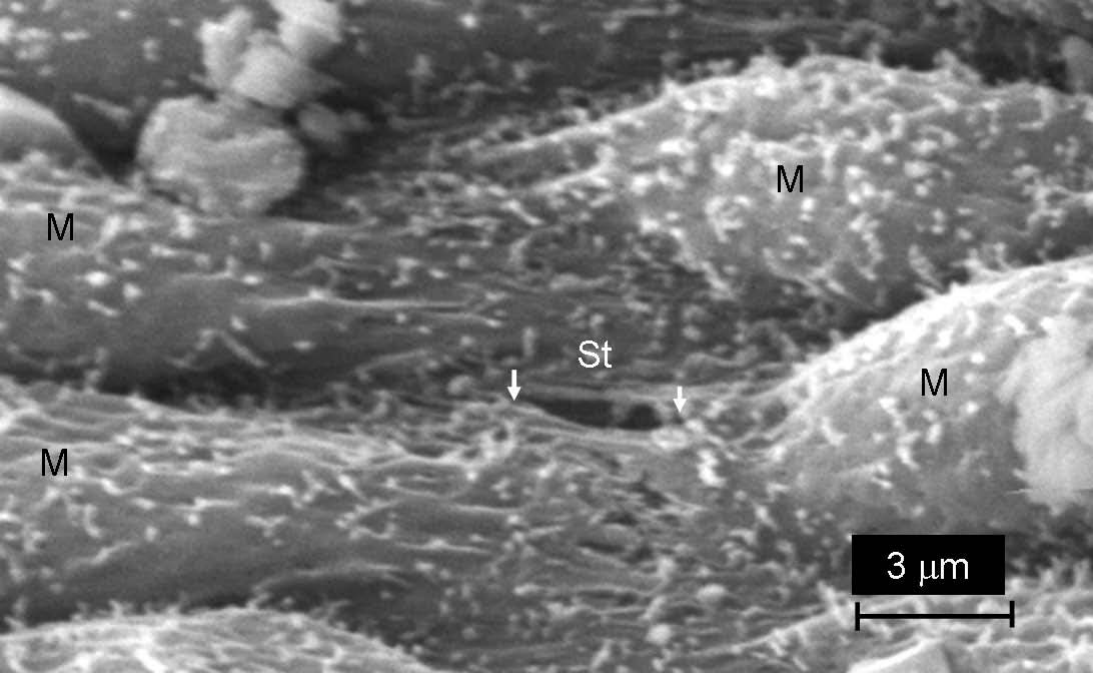
**Scanning electron micrograph image of chest wall from a normal rat showing the parietal pleural surface with mesothelial cells (M) and a stoma (white arrows, St) that is approximately 3 μm in diameter**.

The visceral and parietal mesothelium are both composed of a single layer of mesothelial cells, a basal lamina of connective tissue and a loose connective tissue layer with blood and lymph vessels. Mesothelial cells have several functions in normal pleural action [35]. Pleural fluid is constantly produced by hydrostatic pressure from the sub-pleural capillaries [35], supplemented by glycoproteins secreted by the mesothelial cells [36]. The pleural fluid and its constant outflow (see below) maintains tight coupling of the lungs to the chest wall, allowing diaphragmatic muscle contraction and relaxation to expand and passively relax the lungs during breathing movements. The pleural space is a narrow, variable space, that is up to 20 μm or so in sheep rapidly fixed at death and presumed to be similar in size in humans [35]. The pleural fluid turns over rapidly [37], continuously exiting through stomata in the parietal (not the visceral) pleura via lymphatic capillaries; these stomatal openings on the parietal pleural surface are between 3 and 10 μm in diameter (Figure 8). They are often found in association with 'milky spots', large accumulations of leukocytes present on the parietal pleura [38] and presumed to be involved in immune activity in the pleural space. The pleural fluid outflow through these stomata drains to the lung lymph nodes in the region of the mediastinum and this pathway is important in the clearance of particles and fibres that reach the pleural space (see below). The drainage of fluid from the pleural space carries particles in the lymph to the hilar lymph nodes, mediastinal lymph nodes, parasternal lymph nodes and posterior mediastinal lymphoid tissue [39]. The stomata are most densely situated in the most caudal and posterior intercostal spaces although and are more lightly scattered in more cranial and anterior intercostal regions [40].

## A fraction of all deposited particles transit through the pleura, exit via the stomata and form 'black spots' around the stomata

Applying the classical dose/response toxicology paradigm to the unique pleural pathology seen with asbestos and other fibre exposures, it may be assumed that since the response occurs at the pleura, the dose must be applied at the pleura. It might be argued therefore that since fibres produce pleural pathology whilst particles do not, fibres must reach the pleura and particles must not. However, a body of literature exists to the effect that in fact a proportion of **all **deposited particles reach the pleura, pass through the pleural space and exit through the stomata. In the process of this they elicit range of low to higher grade responses there in the form of parietal pleural 'black spots'.

Evidence that all particles pass through the pleura comes from a substantial literature concerning the almost universal existence of these "black spots", observable on the parietal pleural wall at autopsy. These mark the stomata and arise where particles must focus to exit the pleural space at the stomata and where they enter the sub-mesothelial connective tissue around the stomatal mouths. In the study by Mitchev *et al*. [41], 150 consecutive necropsies of urban dwellers were examined in Belgium. Of the 96 male and 54 female necropsies, whose age ranged from 22-93 years, black spots were almost invariably seen (>90% of autopsies) on the parietal pleura. The authors noted that their location appeared to be related to the structures responsible for the lymphatic drainage of the pleural cavity and they considered these to mark the points of pleural fluid resorption. Black spots were also present on the pleural face of the diaphragm, suggesting that there is pleural fluid outflow in a caudal direction. The black spots in the Mitchev study of normal individuals at autopsy clearly reflects that deposited soot particles normally pass through the pleura some of them accumulating in the parietal pleural wall forming black spots. The black spots contain particles and elicit a tissue response which is a low grade in city dwellers, where there is accumulation of dust-laden macrophages and lymphocytes. In coal miners, however, with their large exposures to particles, the mixed dust particles trigger a higher-grade inflammatory reaction of the parietal pleura with concomitant low grade fibrosis in the 'black spots' which are very pronounced [42]. Occasionally pleural inflammatory reactions to interstitialisation of the mixed dust at the black spots are more pronounced, producing more severe granulomatous structures with concentrically arranged collagen fibres [42]. In one study [42], 12 patients with black spots (8 at autopsy and 4 surgically) who were largely miners, had their black spots removed and sectioned for histological purposes. As might be expected with such high dust exposure, the black spots were extremely well-demarcated and followed the lines of lymph flow across and through the parietal pleura.

The most severe and frequently-documented example of pleural response to dust is asbestos pleural plaques. Pleural plaques are commonly seen in asbestos-exposed individuals occurring only on the parietal pleura and diaphragm as discrete, raised, irregularly shaped areas a few millimetres to 10 centimetres in size, having a greyish to ivory white colour depending on their thickness [43]. It is important to note that pleural plaques occur in greatest profusion in exactly the sites where the stomata are in greatest profusion i.e. pleural plaques are '...most commonly found on the posterior wall of the lower half of the pleural spaces, those in the intercostal space tended to have an elliptical shape and ran parallel to the ribs above and below...'. On histological section plaques can be seen to be composed of dense bands or weaves of avascular and largely acellular collagen, with only the occasional fibroblast nucleus to be seen; they are sometimes calcified [44]. These collagenous plaques, whilst commonly seen in association with asbestos exposure are not unique to it, being found following pleural infection or trauma and so can be presumed to be the way that the pleura reacts to injury [44].

Thus there is clear evidence that a proportion of all deposited particles, most commonly urban particulate matter, reach the parietal pleura where they may interstitialise around the stomata and elicit responses. The severity of the response is dependent on the intrinsic toxicity of the dust, with increasing levels of inflammatory/fibrotic response as follows:- soot < mixed mineral dust < short asbestos. The benign nature of asbestos pleural plaque-type responses is evident in the lack of reports of mesothelioma in city dwellers or coalminers despite the prevalence of black spots in these populations and the notable lack of asbestos pleural plaque progression to malignancy [45]. Since normal asbestos pleural plaques are benign and not pre-cancerous, we hypothesise that pleural plaques are a special case of a 'black' spot caused by short asbestos fibres which elicit an unusually florid collagenous response, or as a result of a very high dose of short fibres reaching the peri-stomatal wall. The emphasis on shortness here is important since the key feature of black spots, we contend, is that the particles and short fibres are small enough to negotiate the stomatal openings where they mostly clear to the lymph nodes whilst some interstitialise into the sub-mesothelial interstitium through the proximal lymphatic capillary walls. As described below, this contrasts with events that may occur with long fibres; these cannot negotiate the stomata leading to retention at the stomatal openings, initiating a very different pathobiological sequence of events culminating in a different pathological outcome.

Our knowledge regarding the pathway by which particles reach the pleura from the lung parenchyma is well summed up in a recent review '... How asbestos fibres that have impacted the airway wall migrate to the pleural surface ..... is quite obscure. ..' [1]. Lymphatic flow from the parenchyma to the pleural space is one obvious possibility [46] but such a pathway, if it exists, is not well-documented [1]. A fluid dynamics model of fibre translocation highlights two possible pathways [47], the first of these being by normal lymph flow centrally to the mediastinum and then into the blood via the thoracic duct followed by extra-vasation in the pleural capillaries during the formation of pleural fluid. This rather tortuous route disregards the filtering role of the lymph nodes and seems to us to be intuitively unlikely. The second route requires inflammation in the parenchyma, caused by the fibres, to reverse both the normal flow of lymph and the normal trans-pleural pressure, resulting in a net flow of fluid and fibres directly into the pleural space from the underlying parenchyma [47]. This latter process cannot be the explanation for normal transit of particles to the pleura that gives rise to black spots (see above) in normal people, who have no pulmonary inflammation. Therefore, even if such an inflammation-dependent pathway exists, a pathway that is independent of inflammation clearly operates for compact particles in normal people. Further research is needed to establish the mechanism of transport of fibres to the pleural space.

## Long fibre retention at the stomata of the parietal pleura

**Figure 9:**
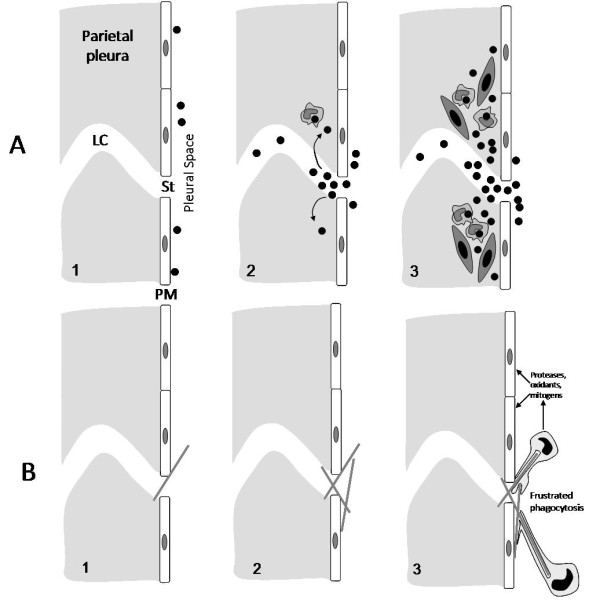
**A) diagram showing the events leading up to formation of black spots**. A1) particles enter the pleural space; A2) in focusing to exit via the stoma (St) some particles interstitialise through the loose lymphatic capillary endothelium and macrophages begin to accumulate in response; A3) macrophages and particles form a mature 'black spot' with mesenchymal cell activation and proliferation depending on the toxicity and dose of the particle. B) In B1 a single long fibre is intercepted as it attempts to negotiate the stomatal opening and is retained; 2) other fibres are caught up and there is an accumulation of retained long fibres; 3) macrophages attempt to phagocytose the fibres and undergo frustrated phagocytosis releasing a range of pro-inflammatory, genotoxic and mitogenic mediators close to the pleural mesothelial cells. PM = pleural mesothelium; St = stoma; LC = lymph capillary.

So from the above there is good evidence to support the contention that a fraction of all deposited particles reach the pleura by an obscure pathway and that short fibres and compact particles leave the pleura through the stomatal openings. Most of the particles are transported to lymph nodes and some enter the interstitium at the mouth of the stoma to form a 'black spot' or equivalent. Long fibres that reach the pleural space, however are an exception to this, since they have the potential to physically block the stomata due to their difficulty in negotiating the bend into the stoma which would result in interception of the ends of the fibre with the walls of the stomatal openings and with the lymph vessels walls themselves. This is likely to lead to mesothelial and endothelial cell damage at this site, inflammation and the accumulation of pleural macrophages attempting to phagocytose these retained fibres. The macrophages are likely to undergo frustrated phagocytosis in attempting to enclose the long fibres and so release cytokines and oxidants. This would lead to further inflammation, fibrosis and genotoxicity in the bystander mesothelial cells in these areas of congestion around the stomatal entrances. Direct interaction between retained long fibres and mesothelial cells around the stomata could also result in direct genotoxicity. Eventually some stomata are likely to be entirely blocked by cells and fibres. Figure 9 demonstrates diagrammatically the difference between formation of black spots and pleural plaques with particles and short fibres (A), compared to the response to long fibre retention at parietal stomata (B).

This means that that the primary lesion caused by long fibres must form at the parietal pleura, the site of retention of long fibre dose and therefore the site of response. Mesothelioma would therefore originate not at the visceral pleura but at the parietal pleura. There is good evidence to suggest that this is indeed the case, and numerous studies using thoracoscopy have confirmed that the origin of mesothelioma is the parietal pleura [6]. This is reflected in the staging of mesothelioma which recognises that early mesothelioma is confined to the parietal pleura, while more advanced mesothelioma involves the visceral pleura [48]. Indeed, the prognosis for mesothelioma when it only involves the parietal pleura is much better by around 30 months, than the prognosis arising when mesothelioma involves visceral pleura [6]. This reflects the earliness of the disease stage when it is still confined to the parietal pleura. From a toxicological viewpoint this means that the focus of attention in trying to determine whether any fibre is likely to cause mesothelioma should not be focussed on the question 'Do fibres reach the pleura?' but should be focussed on the question 'Are the fibres retained in the parietal pleura?'.

Fibres found in digested human lung and visceral pleura at autopsy following death from mesothelioma are often short [49] but, as described below, these are not the site to seek the effective fibre 'dose' for mesothelioma, since the parietal pleura is the site of mesothelioma initiation. In fact the site where the effective dose for long fibres is initially applied is the parietal mesothelium, which has seldom been sampled for fibre burden or dimensions. However, in several studies, fibres recovered from the parietal pleura have also been found to be short [50,51]. This may be explained by the fact that, as described above, the location of the longer fibres is likely to be very focal, at the stomata. When this area was specifically sampled in 14 patients diagnosed with asbestos-associated diseases, including mesothelioma, much longer fibres were found concentrated there [5].

Correct sampling is key to determining the important measure of dose and since this is likely to be found only in 'hot-spots' at stomata but these are heterogeneously distributed across the parietal pleura, are very small and so difficult to sample.

## Fibre biopersistence and the hypothetical model

In the case of non-biopersistent fibres, the degree of their biopersistence, specified by the retention half-time, will dictate the likelihood that they will reach the pleura and the impact that they will have there. For fibres of very low biopersistence such as the chrysotile fibres with half-lives of around 1 day [52] it seems likely such chrysotile fibres undergo dissolution and breakage in the lung parenchyma in the hours following deposition, such that no long fibres are likely to reach the pleura; only short fibres, if any fibre-like structures remain at all, are likely to transit to the pleural space. In the case of fibres that are moderately biopersistent, the long fibres may retain their structure en route to the pleural space, all the while undergoing dissolution/breakage. If long fibres are sufficiently biopersistent to retain their fibrous structure long enough to enter the pleural space they may be retained at the parietal stomata, initiating frustrated phagocytosis and granuloma formation. Depending on the extent of their biopersistence, however, fibres could still dissolve and break within the macrophages as a result of the high pH within the phagolysosomes, allowing the granuloma to resolve. The exact tempo of translocation of particles and fibres to the pleural space is unknown, but in less than 1 day following inhalation of short, essentially particulate, CNT in mice, the CNT were evident in the sub-pleural extracellular matrix [53]. This suggests that compact particles or very short HARN may reach the pleural space rapidly following deposition; however such short HARN and compact particles are not likely to be retained at the stomata. No inhalation study with long CNT has yet been carried out but fibres long enough to be retained at the parietal stomata may move more slowly through the parenchyma, to the pleura, because of their greater dimensions causing 'drag' to their movement through fluid. Research is needed to elucidate the relationship between fibre length and biopersistence in leading to pleural transport, mesothelial injury, inflammation and mesothelioma.

## Carbon nanotubes in the pleura

**Figure 10:**
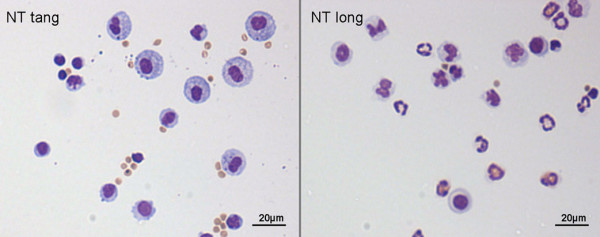
**Cytospin preparations of pleural lavage cells from mice treated with short/tangled CNT (left) and long CNT (right)**. Arrows indicate granulocytes. Note PMN and eosinophils (arrows) indicative of inflammation in pleural leukocytes from rats exposed to the long NT only.

We approached the issue of the potential mesothelial toxicity and pleural toxicity of carbon nanotubes by first attempting to determine whether, similar to asbestos, carbon nanotubes showed length-dependent toxicity to the mesothelium. Based on the above argument we also hypothesise that the long CNT would be retained at the parietal pleural around the stomata. In early studies we used the peritoneal mesothelium lining the peritoneal cavity as the target mesothelium. These studies demonstrated that there was indeed length-dependent inflammogenicity and fibrogenicity of carbon nanotubes in the peritoneal cavity, mimicking asbestos [2]. As predicted from the fibre pathogenicity paradigm, the key length appeared to be between 15-20 μm, the length beyond which macrophages cannot stretch and enclose fibres, thus eliciting frustrated phagocytosis. In the follow up to the studies we utilised the pleural mesothelium and developed a model of injection of nanotubes and asbestos into the pleural space of mice. This can be affected quickly in non-anaesthetised mice using a very fine needle with a collar at the level of the bevel in the needle to restrict penetration through the chest wall allowing injection only into the pleural space. Following injection, the injectate distributes through the pleural space as was evident by inspection of lungs immediately following injection. In these studies we injected the same panel of fibres used in the peritoneal cavity in the Poland studies, i.e. long and short amosite asbestos samples, two long nanotubes samples and two short/tangled nanotubes samples and nanoparticulate carbon black as a graphene control. Following injection the pleural cavity was lavaged to determine the inflammatory response. We found clear evidence of length-related inflammation in the pleural space with both the long amosite and the two long nanotubes samples causing inflammation while all the other short samples failed to elicit significant inflammation (Figure 10).

**Figure 11:**
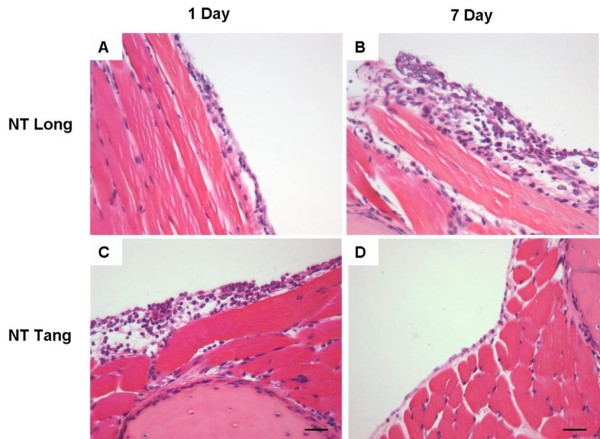
**Histological sections through the chest wall of mice treated with long straight nanotubes (A, B) or short, tangled nanotubes (C, D) for 1 or 7 days**. Note the progressive thickening of the mesothelium in A and B in response to long straight nanotubes; this contrasts with the acute thickening of the pleural layer with short/tangled CNT which shows resolution to normal mesothelium by 7 days (D). Scale bars represent 20 μm.

In a time course, the long nanotubes caused a sustained high level of inflammation at 7 days, which was the same as was present at 1 day. This is in contrast to the events in the peritoneal space where there is a decline in inflammatory response over 7 days to levels of about one-fifth present on day 1 by day 7. Based on our hypothesis that long fibres were retained at the parietal pleural and that short fibres were not, we used paraffin wax histology to examine the parietal pleural surfaces. In keeping with the hypothesis, long fibres which were visible on day 1 following injection were still visible in granulomas at the surface of the parietal pleura on day 7 (Figure 11). No short fibres were visible in sections of parietal pleura at 1 or 7 days, however the activated, thickened mesothelium seen at day 1 had returned to normal by day 7 suggesting that the short fibres had been cleared (Murphy, F., Poland, C.A., Ali-Boucetta, H., Al-Jamal K.T., Duffin, R., Nunes, A., Herrero, M-A., Mather, S. J., Bianco, A., Prato, M., Kostarelos, Donaldson, K. Long but not short nanotubes are retained in the pleural space initiating sustained mesothelial inflammation. Submitted for publication).

**Figure 12:**
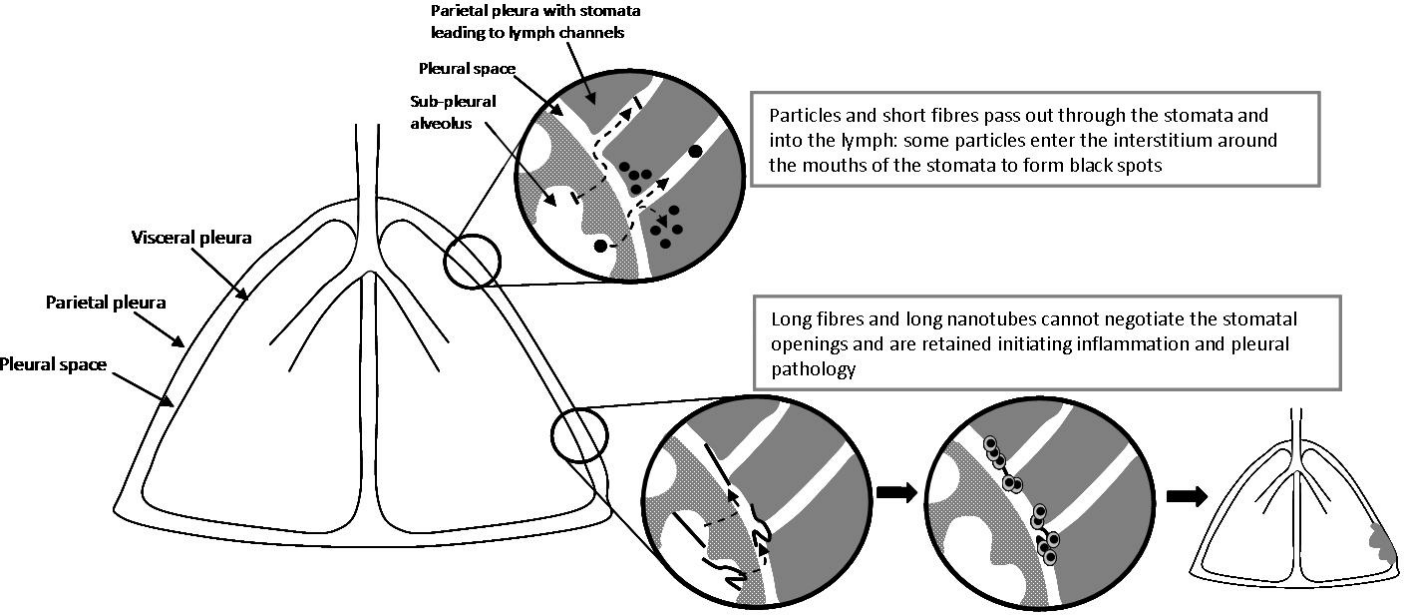
**Hypothesised sequence of events leading to pleural responses as a consequence of long fibre retention at the parietal pleural stomatal openings**.

We therefore hypothesise that the retention of long fires at the stomatal openings on the parietal pleura, coupled with frustrated phagocytosis of pleural leukocytes that attempt to ingest them, produce a chronic pleural mesothelial inflammatory response. Chronic inflammation is known to be a driver for proliferation, genotoxicity, growth factor synthesis and release that are likely to culminate in pathology such as fibrosis, pleural effusion and mesothelioma (Figure 12).

## Implications for testing of high aspect ratio nanoparticles (HARN)

The foregoing discussion has highlighted the importance of the parietal pleura as the target for the long fibre hazard following pulmonary deposition and the site of initiation of mesothelioma. In addition to carbon nanotubes, there are a whole new generation of high aspect ratio nanoparticles (HARN), such as nanorods and nanowires. These are made of a wide range of materials, including silica, silver, nickel and various forms of carbon. There is a need to test these materials and understand their potential for causing mesothelioma. Mesothelioma has a very long latent period and in rats, following inhalation of asbestos, mesothelioma commonly does not develop until near the end of life in a small proportion of animals. The peritoneal cavity has been used as a more efficient model for mesothelioma induction but has been criticised because of its non-physiological nature and irrelevance for risk assessment. However, the peritoneal cavity does show size-restricted clearance and subsequent sensitivity to retained long fibres. An appreciation of the role of the parietal pleura as the site where fibres are retained leading to pleural pathology of various sorts which accompany exposure to fibres, means that a rational testing strategy could attempt to identify early changes in this tissue following fibre exposure. New techniques that allow investigators to home in on specific areas, eg laser capture, would enable the areas of the parietal pleura where the stomatal openings occur to be identified and studied in detail for the presence of fibres and their molecular consequences. Thus there is the prospect of studying oxidative stress, inflammation and genotoxicity at an early stage in the very target tissue where mesothelioma is likely to arise. This should revolutionise the ability to screen for pathogenic fibres amongst emerging HARN and allow us to look anew at the effects of asbestos and more conventional fibres that affect the pleural mesothelium and we look forward to future studies that utilise this knowledge.

## Implications for asbestos and mesothelioma

The emphasis on the parietal pleura as the site of retention and focus for the long fibre dose has important implications for our understanding of the origins of mesothelioma, a subject of considerable scientific and, arguably, even greater medico-legal significance. Mesothelioma continues to be a global problem due to ongoing exposure to fibres and as a legacy from past exposure to asbestos, even in countries where asbestos is currently banned or has been regulated out of use for decades [54]. Lung tissue burdens of asbestos fibres have long been used as an index of exposure, but the above discussion highlighting the parietal pleura, not the lung tissue, as the site of origin of mesothelioma calls into question the relevance of parenchymal lung fibre burdens as a correlate of mesothelioma. The **lung **diseases caused by asbestos i.e. lung cancer and asbestosis - may well be related to the lung parenchyma tissue burdens, since one would reasonably look in the target tissue for the effective dose. However the same logic would dictate that the effective dose for mesothelioma, which arises in the parietal pleura, should be sought in that tissue. In fact the parietal pleura fibre burden has been studied, but only very occasionally; for example Dodson and Atkinson [55] cite Sebastien [56] as stating that, for asbestos fibre burden "lung parenchymal retention is not a good indicator of pleural retention: indeed, there was no relationship between parenchymal and pleural concentrations". This would be predicted from the arguments presented in this paper. Therefore, whilst the lung parenchyma is a site of fibre accumulation that is likely related to exposure, the lung parenchyma is not expected to focus the effective dose for mesothelioma in the way that the parietal pleura does [5] through its action as a kind of 'sieve' that selectively retains long fibres.

Even supposing the parietal pleura were to be chosen as the tissue of choice for assessing effective dose of long fibre for the mesothelioma hazard, a considerable problem is posed in sampling it for fibre-burden analysis because of their small size and the heterogeneous distribution of the stomata over the parietal pleura. Yet these are exactly the sites that should be sampled in order to find the dose responsible for the mesothelioma response or to sample the site of developing mesothelioma in order to determine its molecular ontogeny. These 'hot-spots' of dose could not be easily selected at autopsy by a pathologist unless they knew specifically where to look and even then the diluting effect of non-stomatal tissue in the immediate vicinity could easily confound any attempt to determine the specific long fibre dose at the stomata.

## Conclusion

We have reviewed the evidence for the hypothesis for the behaviour of long fibres in the parietal pleura, focusing on nanotubes as a new potential pleural hazard, although the discussion is relevant to all fibres including asbestos. We argue from existing evidence that a fraction of all deposited particles reach the pleura and from evidence on the mechanism of particle clearance from the pleura, to argue that the parietal pleura is the site of retention of long fibres (Figure 10). We suggest that their retention there, a consequence of length-restricted clearance through the normal stomatal clearance system, initiates inflammation and pleural pathology including mesothelioma. We cite data from thoracoscopy to support the contention that, as would be anticipated from the foregoing, the parietal pleura is the site of origin of pleural mesothelioma. This general hypothesis on the key role of fibre length-restricted clearance from the pleural space as a mechanism for delivering a high, focussed, effective dose of long fibres to the mesothelial cells around the parietal pleural stomata, has important implications. These lie in future research into the mesothelioma hazard from HARN but also for our current view of the origins of asbestos-initiated pleural mesothelioma and the use of lung parenchymal fibre burden as a correlate of this tumour, which arises in the parietal pleura, not the lung parenchyma or visceral pleura.

## Competing interests

The authors declare that they have no competing interests.

## Authors' contributions

KD Drafted the manuscript and provided background material for inclusion. CP Provided important input on the hypothesis and the data described. RD Provided key input in the review of literature and its interpretation.  FM Provide important data and background and developed the hypothesis. All authors read and approved the final manuscript.

## Acknowledgements

We gratefully acknowledge the financial support of the Colt Foundation (KD, CAP, RD) and the UK Department of Health (FM)
